# Use of traditional and complementary medicine by ethnic Indian women living with polycystic ovary syndrome: a global survey

**DOI:** 10.1186/s12906-023-04229-9

**Published:** 2023-11-03

**Authors:** Vibhuti Samarth Rao, Mike Armour, Birinder S Cheema, Caroline A Smith, Lisa Moran, Romain S Perera, Siew Lim, Sabrina Gupta, Michael De Manincor, Rama Vaidya, Carolyn Ee

**Affiliations:** 1 NICM Health Research Institute, Western Sydney University, Penrith, NSW 2571 Australia; 2 School of Health Sciences, Western Sydney University, Campbelltown, NSW 2560 Australia; 3 Monash Centre for Health Research and Implementation, Monash University, Clayton, VIC 3168 Australia; 4 Department of Cell and Developmental Biology, University College London, London, WC1E 6BT UK; 5 Health Systems and Equity, Eastern Health Clinical School, Monash University, Boxhill, VIC 3128 Australia; 6 School of Psychology and Public Health, La Trobe University, Melbourne, VIC 3689 Australia; 7 Division of Endocrine and Metabolic Disorders, Kasturba Health Society’s Medical Research Centre, Mumbai, 400056 India

## Abstract

**Background:**

Traditional, complementary, and integrative medicine (TCIM) is commonly used by those living with Polycystic Ovary Syndrome (PCOS) but little is known about the use of TCIM such as yoga and Ayurveda in ethnic Indian women with PCOS living worldwide. This survey aimed to explore the prevalence and types of TCIM used and in particular the pattern of use of yoga and Ayurveda including reasons for not using and future interest in using them among non-users.

**Method:**

An online, international cross-sectional survey was conducted using a pre-designed survey tool. Participants were ethnic Indian women of reproductive age who reported that they were medically diagnosed with PCOS. Descriptive analysis was used to identify the proportion of TCIM users, while a multivariable binary logistic regression was used to analyze their characteristics.

**Results:**

Data from 3130 respondents were analysed. The prevalence of TCIM use was 80% (2515/3130). Yoga and Ayurveda were the most frequently practised TCIM modalities with a prevalence of 57% and 37% respectively. We found a high future interest in using yoga (81%) and Ayurveda (70%) among the non-users. The motivation for most Ayurveda use was a recommendation from family/friends (66%), rather than personal choice (38%) or the internet (19%). Most women used Ayurveda because it has natural ingredients (64%) and it is safe (60%) and cited its use to be safe and somewhat helpful. The majority of women were currently practising yoga (73%) up to four times a week (54%) at home (93%). Yoga was primarily used to improve overall health (67%), manage weight (64%), stress (54%) hormonal imbalance (49%) and emotional well-being (48%). Barriers to practising yoga were common among the current users and non-users and included lack of motivation (59% and 59%), time constraints (48% and 39%), and non-availability of yoga teachers specialised in PCOS (31% and 23%). Most women found yoga to be helpful and preferred individual one-on-one (52%) yoga sessions specifically tailored for PCOS (58%).

**Conclusion:**

This is the first international study that discovered the prevalence and pattern of TCIM use among ethnic Indian women with PCOS living worldwide. We support the urgent need for more research, education, and regulation of different TCIM modalities to promote safe and effective practices globally.

**Supplementary Information:**

The online version contains supplementary material available at 10.1186/s12906-023-04229-9.

## Background

Polycystic Ovary Syndrome (PCOS) is a chronic endocrine disorder of unknown origin in women of reproductive age, characterised by irregular menstruation, hyperandrogenism and/or polycystic ovarian morphology on ultrasound [1]. It is heterogeneous, of complex pathogenesis and can adversely affect women’s reproductive, metabolic, psychological and emotional health [2]. PCOS is highly prevalent among Indian women with an estimated prevalence of up to 22% [3]. Despite continuous efforts to develop effective treatments, women with PCOS are burdened by the complex symptoms and lifelong morbidities associated with PCOS and seek alternate ways such as Traditional, Complementary and Integrative Medicines (TCIM) to manage their symptoms [4–7].

In general, a large proportion of the world’s population depends on various forms of TCIM for their primary health needs [8]. Nearly 50–70% of people living in developed countries and 40–80% of people in developing countries now use some form of TCIM [9, 10]. The growing evidence for and increasing use of TCIM indicates that it might be useful for the prevention and treatment of chronic diseases [8, 11, 12]. Various TCIM modalities particularly yoga, mindfulness, acupuncture, traditional Chinese medicine, Ayurveda, nutraceuticals, and herbal medicine have been studied for their potential benefits in the treatment of PCOS [13–16]. However, little is published about the prevalence and use of TCIM in women with PCOS, especially among ethnic Indian women where TCIM use is generally higher when compared to other population groups [17]. Traditional medicines are part of a broader belief system and an integral part of everyday lifestyle among Indian communities. Moreover, there is a pluralistic healthcare system in India where along with conventional medicine, several other TCIM modalities (Ayurveda, Yoga, Unani, Siddha, and Sowa-Rigpa, along with Homeopathy- termed as AYUSH systems) are institutionally recognised and practised for several years [18].

Ayurveda and yoga are TCIM modalities that originated in the Indian sub-continent for which there is preliminary but promising evidence of their role in managing symptoms of PCOS [16, 19]. Ayurveda uses multifaceted diagnostic and treatment measures, which include complex and individually tailored interventions; bio-purification therapies called *Panchakarmas*; lifestyle recommendations for dietary, behavioural and other activities such as seasonal and daily regimens and use of single/polyherbal compounds [20]. Yoga is a sister science of Ayurveda and a mind-body practice that encompasses many aspects including *asana* (physical postures), *dhyana* (meditation), and *pranayama* (breathing techniques) [21]. Globally, there are several styles/schools of yoga practices such as *Astanga* and *Hatha* yoga [21]. The practice of yoga generally aims to integrate body and mind and bring positive behavioural changes holistically, considering individual preferences [22]. Furthermore, a few systematic reviews and meta-analyses have demonstrated the promising role of yoga in improving treatment outcomes and reducing symptoms in women with PCOS [19, 23].

Despite high interest in the use of TCIM, evidence of its use in PCOS is limited. We found only one study published in 2012 that explored the use of TCIM in a cohort of women with PCOS living in Australia [7]. However, the ethnicity of the sample was not reported. We know that ethnic Indian women use TCIM irrespective of their geographical location to manage their health and diseases [24–31]. Thus, it is reasonable to speculate comparable trends of TCIM use in ethnic Indian women with PCOS, due to the deeply rooted culture and clearer institutional recognition of TCIM. We found no previous study that used a large, international community sample to investigate the prevalence of TCIM and patterns of use of Ayurveda and yoga in ethnic Indian women with PCOS, including those living outside India. With the increased interest of women in holistic healthcare, it is important to understand what TCIM approaches ethnic Indian women living with PCOS are using and specifically their indigenous health systems such as yoga and Ayurveda, to ensure that ongoing and future PCOS care includes culturally appropriate elements.

### Aim and objectives

The primary aim of this study was to explore the prevalence and use of TCIM in ethnic Indian women with PCOS living worldwide. The objectives were to explore and examine: (i) the prevalence, types, and predictors of TCIM use; (ii) the pattern of use of Ayurveda and yoga including perceived effectiveness and self-reported adverse events related to the use of Ayurveda and preference for yoga; (iii) reasons for not using Ayurveda and yoga, and future interest in using them among the current non-users.

## Methods

This paper is part of a larger data set collected via an online survey of 4409 ethnic Indian women with PCOS [32]. This paper specifically reports on the use of TCIM, including yoga and Ayurveda. In this cross-sectional online survey, our research team of multidisciplinary experts (a general practitioner, an Ayurveda practitioner, yoga instructors, dietitians, an exercise physiologist, and women’s health experts) developed the survey tool. VR piloted the online anonymous survey tool using Qualtrics software (Qualtrics Ltd. Provo, UT, USA) with consumers (two women with PCOS living outside India and three living in India) and changes were made to a few questions for a better understanding. The tool consisted of open- and closed-ended, and multiple-choice questions that captured demographic features, clinical features including co-morbidities, family history, and forms of TCIM- which were modified, however, were based on international complementary and alternative medicine questionnaire, (I-CAM-Q) [33], patterns of use of yoga and Ayurveda including the perception of effectiveness and safety. To understand the perceived effectiveness of Ayurveda and yoga, a recommendation scale ranging from 0 to 10 (with 0 being not recommended and 10 being extremely recommended) was used. A copy of the survey instrument can be found in Supplemental File 1. The survey took approximately 30 min to complete. Participation was voluntary and features were enabled within Qualtrics that prevented multiple completions from either a single internet protocol (IP) address or the same computer.

We used a wide range of recruitment strategies to include ethnic Indian women living worldwide, such as snowball recruitment through personal and professional connections of the research team, paid social media advertisements, and posting to various PCOS social media advocacy and support groups, Indian women’s groups, and Indian migrant groups (including on Facebook, Twitter, and Instagram) internationally. Eligibility criteria were women of self-reported Indian ancestry (ethnic Indian women either born in India or have at least one parent/grandparent who was born in India), aged 18–55 years and with a self-reported diagnosis of PCOS by a medical doctor and who were able to read and understand English. Data collection occurred from February to June 2021. This study was approved by the Human Research Ethics Committee at the University of Western Sydney (H14103, 2021). Informed consent (implied) was obtained from all participants before the commencement of the online survey.

### Data analysis

The data were transferred to SPSS, IBM statistics package version 28.0.1.0 for data analysis [34]. The data was cleaned and patterns of missing values were examined. Missing values were only replaced for height and weight data. Normal distribution was assessed. Descriptive statistics were performed for normally distributed continuous variables (means and standard deviations [SD]), non-normally distributed continuous variables (medians and interquartile ranges (IQR)) and categorical variables (numbers and percentages). Relationships between categorical variables related to demographics and clinical factors (age, country of birth, country of residence, education, relationship, occupation, BMI, family history of PCOS and type 2 diabetes (T2D)), were determined using the chi-square or Fisher exact test as appropriate. A P-value of < 0.005 was considered statistically significant. A multivariable binary logistic analysis was then conducted to explore the associations between statistically significant exposure variables and the outcome variable (use of TCIM). Adjusted odds ratio (AOR) and 95% confidence interval (CI) were reported.

## Results

### Demographic and other characteristics of the sample

Of the 4409 eligible responses, a total of 3130 respondents answered the questions related to TCIM and thus were included in the analysis. The mean age was 26.9 (SD = 5.5) years. The majority were aged between 18 and 34 years (90%), born in India (82%), diagnosed by a gynaecologist/obstetrician (85%), married (58%), employed (54%), had at least one co-morbidity (65%) and attained a university degree (91%). One-quarter were currently residing outside of India (26%). Respondents reported a mean weight of 69.5 kg (SD = 15.7) and a mean body mass index (BMI) of 27.0 (SD = 5.7), with three-quarters (75%) having a BMI of 23 kg/m^2^ and above (Table 1). Among women with a history of pregnancy and taking treatments to fall pregnant (282/589, 48%), most of them used TCIM (239/282, 85%). The top three key concerns of PCOS reported by the women were: menstrual irregularities (1971, 63%), difficulty losing weight (1808, 58%) and excess facial hair growth (1451, 46%). Of the women with menstrual irregularities, 83% reported using TCIM.

**Table 1:** Association between participant’s characteristics and the use of TCIM (N = 3130)

Participant’s characteristics ^a^	Total Respondents (N = 3130)	USE OF TCIM^b^			
		No^c^ (n = 615, 20%)	Yes (n = 2515, 80%)	P-value^d^	Multivariable analysis^e^, OR (95% CI), P-value
**Age (years), n (%)**				0.43	
18–24	1199 (38)	245 (40)	954 (38)		0.76 (0.50 to 1.16), 0.21
25–34	1623 (52)	317 (51)	1306 (52)		0.78 (0.55 to 1.12), 0.18
35–55	308 (10)	53 (9)	255 (10)		Ref
**Country of birth**				**< 0.001**	
India	2577 (82)	2083 (94)	494 (89)		1.34 (0.95 to 1.90), 0.09
Outside India	203 (18)	142 (6)	61 (11)		Ref
Missing	350 (11)				
**Country of current residence**				**< 0.001**	
India	2046 (74)	357 (64)	1689 (76)		1.81 (1.43 to 2.28), **< 0.001**
Other countries	734 (26)	198 (36)	536 (24)		Ref
Missing	350 (11)				
**Education**				0.08	
Below undergraduate degree	263 (9)	63 (11)	200 (9)		0.79 (0.55 to 1.13), 0.19
Undergraduate degree	1375 (50)	283 (51)	1092 (49)		0.86 (0.69 to 1.07), 0.17
Postgraduate degree	1142 (41)	209 (38)	933 (42)		Ref
Missing	350 (11)				
**Occupation**				0.19	
Unemployed	256 (9)	53 (10)	203 (9)		0.32 (0.07 to 1.42), 0.14
Employed	1486 (54)	291 (52)	1195 (54)		0.35 (0.08 to 1.52), 0.16
Studying	760 (27)	167 (30)	593 (27)		0.41 (0.09 to 1.81), 0.24
Home Duties	249 (9)	42 (8)	207 (9)		0.29 (0.07 to 1.26), 0.10
Other	26 (1)	2 (0.4)	24 (1)		Ref
Missing	353 (11)				
**Relationship status**				0.24	
Single	1113 (40)	226 (41)	887 (40)		1.79 (0.88 to 3.64), 0.11
Married/in a relationship	1623 (58)	316 (57)	1307 (59)		1.85 (0.92 to 3.71), 0.08
Other	44 (2)	13 (2)	31 (1)		Ref
Missing	350 (11)				
**Family history of type 2 diabetes**				0.72	
No	1742 (56)	346 (57)	1396 (56)		Ref
Yes	1353 (44)	262 (43)	1091 (44)		1.06 (0.87 to 1.30), 0.50
Missing	35 (1)				
**Family history of PCOS**				0.69	
No	2550 (82)	495 (81)	2055 (82)		Ref
Yes	571 (18)	115 (19)	456 (18)		0.97 (0.77 to 1.24), 0.84
Missing	9 (0.3)				
**BMI (kg/m** ^**2**^ **)**					
< 18.0	76 (2)	13 (3)	63 (2)	0.25	Ref
18.0-22.9	711 (23)	131 (21)	580 (23)		0.85 (0.42 to 1.68), 0.64
23.0-24.9	435 (14)	75 (12)	360 (14)		1.02 (0.50 to 2.07), 0.95
≥ 25.0	1908 (61)	396 (64)	1512 (79)		0.77 (0.39 to 1.52), 0.46
**Presence of co-morbidity**					
Yes	2042 (65)	1646 (81)	396 (19)	0.637	
No	1088 (35)	869 (80)	219 (20)		

^a^Independent variable/predictors

^b^Dependent variable

^c^Reference value in multivariable analysis

^d^Chi-square/Fisher’s exact test

^e^Binary logistic analysis adjusted for other factors listed in the table except for the presence of co-morbidity

Significant P-values are marked in bold

### Association between demographic and other characteristics and TCIM use

We found no statistically significant associations between the age, education, relationship, occupation, BMI, family history of PCOS and family history of T2D and use of TCIM (Table 1). However, the country of current residence was significantly associated with TCIM use (p < 0.001). Specifically, women living in India had higher odds of using TCIM compared to women living outside India (AOR = 1.81; 95% CI 1.43 to 2.28; p < 0.001). Although the use of TCIM was significantly associated with the country of birth (p < 0.001) in unadjusted analysis, the use of TCIM was not significantly different among women who were born in India (AOR = 1.34; 95% CI 0.95 to 1.90; p = 0.09) compared to women born outside India in multivariable analysis after adjusting for other exposure variables. Married women, living in India and with a history of taking treatment to fall pregnant had increased odds of using TCIM (1.60; 95% CI, 1.03 to 2.46; p = 0.034) compared to women living outside India, unmarried, and without a history of pregnancy and treatment to conceive (Supplemental File 2, Table 1). We found a statistically significant association between the key concern of menstrual irregularity and the use of TCIM (< 0.001). Furthermore, there were higher odds of using TCIM among these women (1.4 95% CI, 1.16 to 1.71; p < 0.001) (Supplemental File 2, Table 2).

### Use of TCIM and its types

**Fig. 1:**
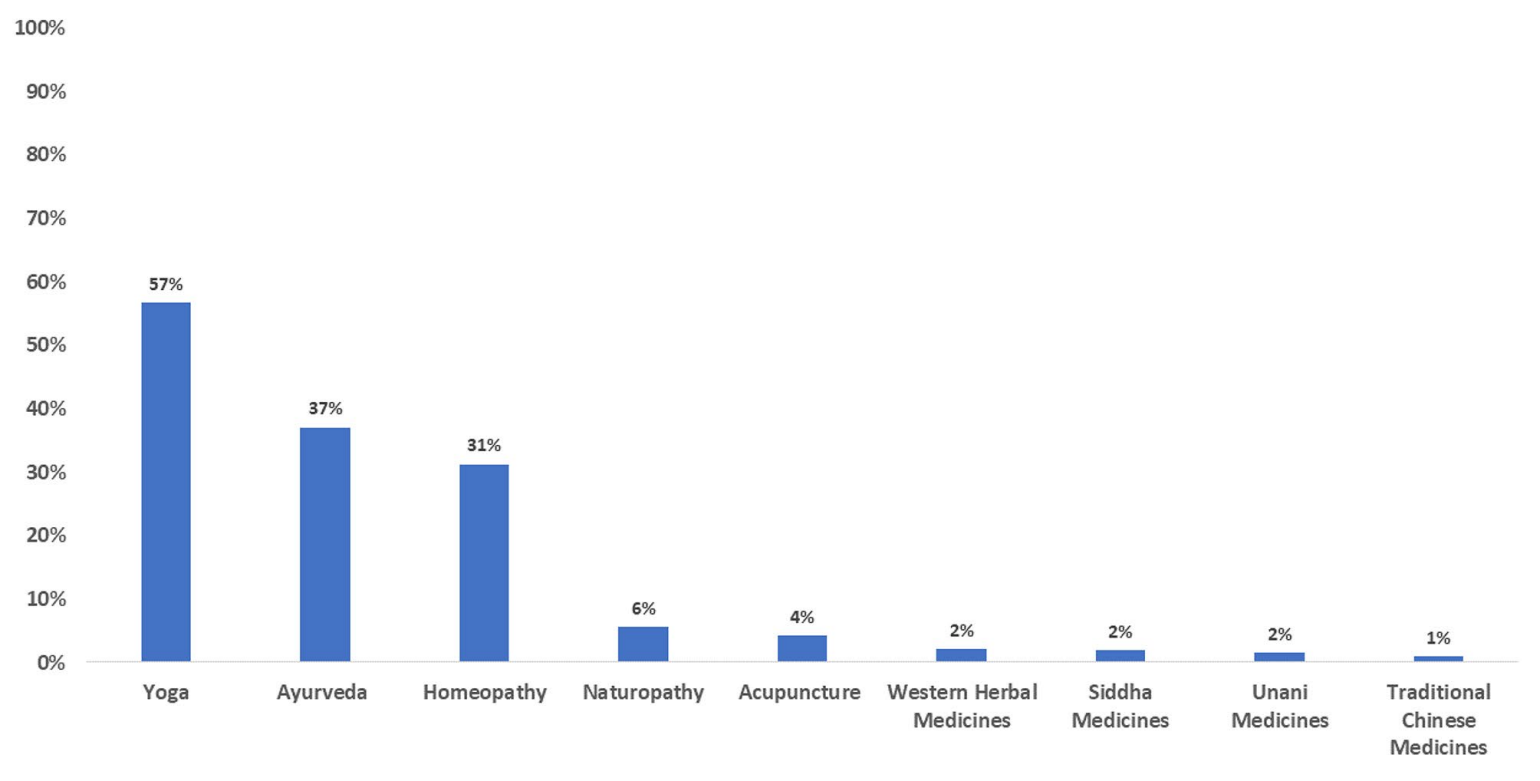
Prevalence of use of TCIM modalities (n = 3130)

More than three-quarters of respondents (2515/3130, 80%) had used at least one form of TCIM to manage symptoms of PCOS. Yoga and Ayurveda were the two most used TCIM modalities with a prevalence of 57% (1775/3130) and 37% (1159/3130) respectively. Other frequently used modalities were homeopathy, followed by naturopathy and acupuncture (Fig. 1). Around half the proportion (52%) used two to three types of TCIM modalities, whereas 43% used only one type of TCIM modality.

### Patterns of use of Ayurveda

As shown in Table 2, more than half the proportion of women (642/1097, 58%) first visited an Ayurveda practitioner more than one year before responding to the survey. However, more than half (663/1113, 60%) started using Ayurveda in the last six months to one year. The most common motivations to use Ayurveda were: family/friends/neighbours/colleagues (733/1113, 66%), personal choice (38%) and the internet (19%).

Among the forms/types of Ayurveda treatments, the use of Ayurveda medicines (946/1083, 87%) was the most common, followed by Ayurveda diet and regimen (34%) and *Panchakarma* (Ayurveda bio-cleansing procedures) (16%) . Almost three quarters (797/1095, 73%) reported not experiencing any adverse events while using Ayurveda, whereas 18% were unsure.

**Table 2:** Pattern of use of Ayurveda among the users (N = 1159)

	Total responses, n (%)
**First visit to an Ayurveda practitioner**,	(n = 1097)
In the last 1–5 years	385 (35)
More than 5 years	257 (23)
In the last 6 months	248 (22)
In the last 1 year	207 (20)
Missing	62 (5)
**Duration of use of Ayurveda**,	(n = 1113)
For the past 6 months	429 (39)
Past 1–5 years	278 (25)
Past 1 year	234 (21)
More than 5 years	172 (15)
Missing	46 (4)
**Frequency of who/what motivated/influenced participants to use Ayurveda**,	(n = 1113)
Family/friends/neighbours/colleagues	733 (66)
Self	419 (38)
Internet	211 (19)
Medical doctor/fertility specialist	58 (5)
Allied health professional (e.g., dietician, exercise physiologist)	51 (5)
Another complementary medicine practitioner (e.g., naturopath, homeopath)	33 (3)
Magazine or newspaper	26 (2)
Television	17 (2)
Missing	46 (4)
**Frequency of forms of Ayurveda used**,	(n = 1083)
Ayurvedic medicines for internal consumption	946 (87)
Ayurvedic lifestyle (e.g., Ayurvedic diet/regimen)	367 (34)
Panchakarma (e.g. Vamana, Virechana, Basti, Nasya)	171 (16)
Ayurvedic medicines for external applications	143 (13)
Missing	76 (7)
**Experienced any adverse events to Ayurveda medicines**	(n = 1095)
No	797 (73)
Unsure	202 (18)
Yes	97 (9)
Missing	64 (6)

**Fig. 2:**
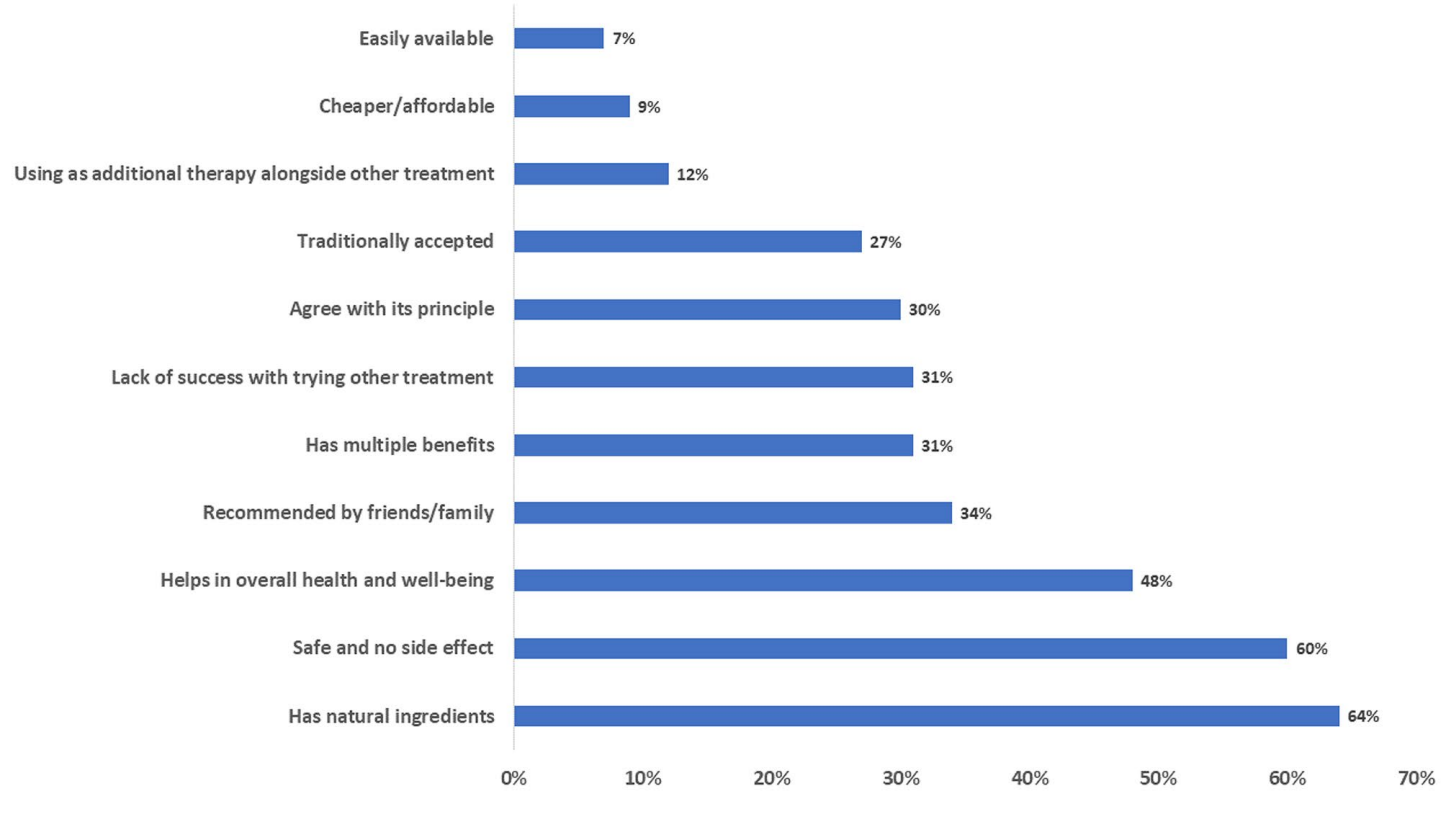
Reasons reported by the participants for using Ayurveda (n = 1113)

The most frequently cited reasons for using Ayurveda were: ‘it has natural ingredients’ (715/1113, 64%), followed by ‘it is safe to use’ (60%); ‘it helps in overall health and well-being’ (48%) (Fig. 2).

Among those who experienced adverse events from Ayurveda (9%), gastrointestinal disturbances and the occurrence of increased body heat were commonly reported. See Supplemental File 2, Table 3 for a list of the adverse events reported by the participants. Women’s self-reported recommendation on a scale of 0–10 showed that Ayurveda was somewhat helpful (7 (IQR = 4)) for managing their symptoms of PCOS (Supplemental File 2, Table 4).

### Reasons for not using Ayurveda & future interest

According to Table 3, among the non-users of Ayurveda (n = 1971/3130), when asked about their reasons for not using Ayurveda, multiple reasons were reported (n = 1926), such as ‘inadequate information’ (39%), ‘uncertainty about effectiveness’ (30%), and ‘not being prescribed by their doctor’ (28%). However, almost two-thirds (n = 1353/1942,70%) expressed their future interest in using it to manage symptoms of PCOS, while 20% remained neutral and 10% were disinterested.

**Table 3:** Reasons for non-utilization of Ayurveda and future interests, (N = 1971)

Frequency of reasons for not using Ayurvedic medicine	(n = 1926)
Not enough information	758 (39)
Not sure if it will work	580 (30)
Not prescribed or recommended by my doctor	542 (28)
Not sure/No definite reason	471 (24)
Lack of scientific evidence	346 (18)
Takes too long to work	304 (16)
Not recommended by family/friends	251 (13)
Not easily available	253 (13)
Not confident to use in conjunction with medical drugs	227 (12)
Smell or taste of the preparations	123 (6)
Not covered by my insurance	65 (3)
Poor quality of herbal medicines	89 (5)
Others than above	94 (5)
Missing	45 (2)
**Interest in trying an Ayurvedic diet or medicines to manage PCOS**	**(n = 1942)**
Interested	1353 (70)
Neither interested nor disinterested	396 (20)
Not interested	193 (10)
Missing	29 (1)

### The pattern of use of yoga and preferences

As shown in Table 4, among the women who practised yoga for PCOS, 48% had started practising yoga within the past six months. When asked about the type/style of their yoga practice, 31% were unsure, 26% reported that they practised whatever type/style was available, and 21% reported practising mixed types/styles.

Among yoga users, 27% (467/1730) reported that they no longer practised yoga. Among those currently practising yoga (73%), most women were currently practising yoga one to four times per week (n = 686, 54%). Home (1178, 93%) was the most used setting to practice yoga and *asana* (physical yoga postures) was the most practised element (1158,93%) followed by *Pranayama* (breathing practices) (832,67%) and *dhyana* (meditation) (569,46%).

When asked about their preferred type/style of yoga practice, approximately half of the women preferred to be taught yoga specifically designed to manage symptoms of PCOS (991/1710, 58%), in a one-on-one (52%) and face-to-face setting (49%).

Most women (988/1707, 58%) did not report any challenges/barriers that prevented them from practising yoga whereas 16% were unsure about this. Of those who identified barriers the most frequently reported were lack of motivation (59%, 401/679), lack of time (48%) and non-availability of the right yoga teacher (PCOS-specific) (31%).

**Table 4:** Patterns of use of yoga, (N = 1775)

	Total responses, n (%)
**Duration of yoga practice**	(n = 1730)
Last 6 months	824 (48)
Last 1 year	381 (22)
Last 1–5 year	311 (18)
More than 5 years	214 (12)
Missing	45 (3)
**Type/style of yoga practice**	(n = 1730)
Unsure about the style	543 (31)
Whatever is available	450 (26)
Mixed style	370 (21)
Traditional Hatha Yoga	208 (12)
Astanga	201 (11)
Power Yoga	166 (9)
Vinyasa	128 (7)
Iyengar	58 (3)
Shivananda Yoga/Yoga Vidya	48 (3)
Bikram /Hot yoga	26 (2)
Kundalini	15 (1)
Krishnamacharya tradition or (Viniyoga)	14 (1)
Other than above	68 (4)
Missing	45 (3)
**Frequency of yoga practice**	(n = 1730)
I do not practice yoga anymore	467 (27)
Currently practising	1263 (73)
Missing	45 (3)
*If currently practising yoga, frequency of the practice-*	(n = 1263)
1–2 times per week	344 (27)
3–4 times per week	342 (27)
5–6 times per week	159 (13)
Twice a month	132 (10)
Daily	116 (9)
Less than once a month	89 (7)
Once a month	81 (6)
*If currently practising, where do you practice*	(n = 1262)
Home	1178 (93)
Yoga studio/school/institute	120 (9.5)
Fitness centre/gym	63 (5)
Park or other public outdoor location	29 (2)
Workplace	11 (1)
Other than the above	8 (1)
Missing	1 (0)
*If currently practising, elements of yoga practice*	(n = 1249)
Asana (physical postures)	1158 (93)
Pranayama (breathing practices)	832 (67)
Dhyana (meditation)	569 (46)
Mantra chanting	200 (16)
Yama/Niyama	28 (2)
Yogic diet	62 (5)
Yogic kriya (e.g. *jala neti, vamana dhouti*)	48 (4)
Other than the above (please specify)	21 (2)
Missing	14 (1)
**Preferred type/style of yoga practice**	(n = 1710)
A yoga class designed to manage symptoms of PCOS	991 (58)
Individual one-on-one	883 (52)
Group practice	501 (29)
General yoga class	377 (22)
Other than the above (please specify)	18 (1)
Missing	65 (4)
**Preferred delivery of yoga instruction**	(n = 1710)
Face-to-face sessions	846 (49)
Anything available/mixed	674 (39)
Pre-recorded online sessions (e.g. Internet, social media, DVD, TV)	566 (33)
Live online sessions	541 (32)
Other than the above (please specify)	17 (1)
Missing	65 (4)
**Barriers to practising yoga**	(n = 1707)
No	988 (58)
Not sure	275 (16)
Yes	444 (26)
Missing	68 (4)
*If yes/unsure- what were those barriers/challenges-*	(n = 679)
Lack of motivation	401 (59)
Lack of time	326 (48)
Cannot find the right yoga teacher (PCOS-specific)	210 (31)
Do not feel flexible enough	181 (27)
Do not feel strong enough	155 (23)
Financial (not enough money to pay for yoga classes)	151 (22)
Do not feel fit enough	150 (22)
Feel embarrassed about my body	136 (20)
Distance (yoga place is far from me)	116 (17)
Physical barriers such as injury	75 (11)
Other than above	44 (6)
Missing	40 (6)

**Fig. 3:**
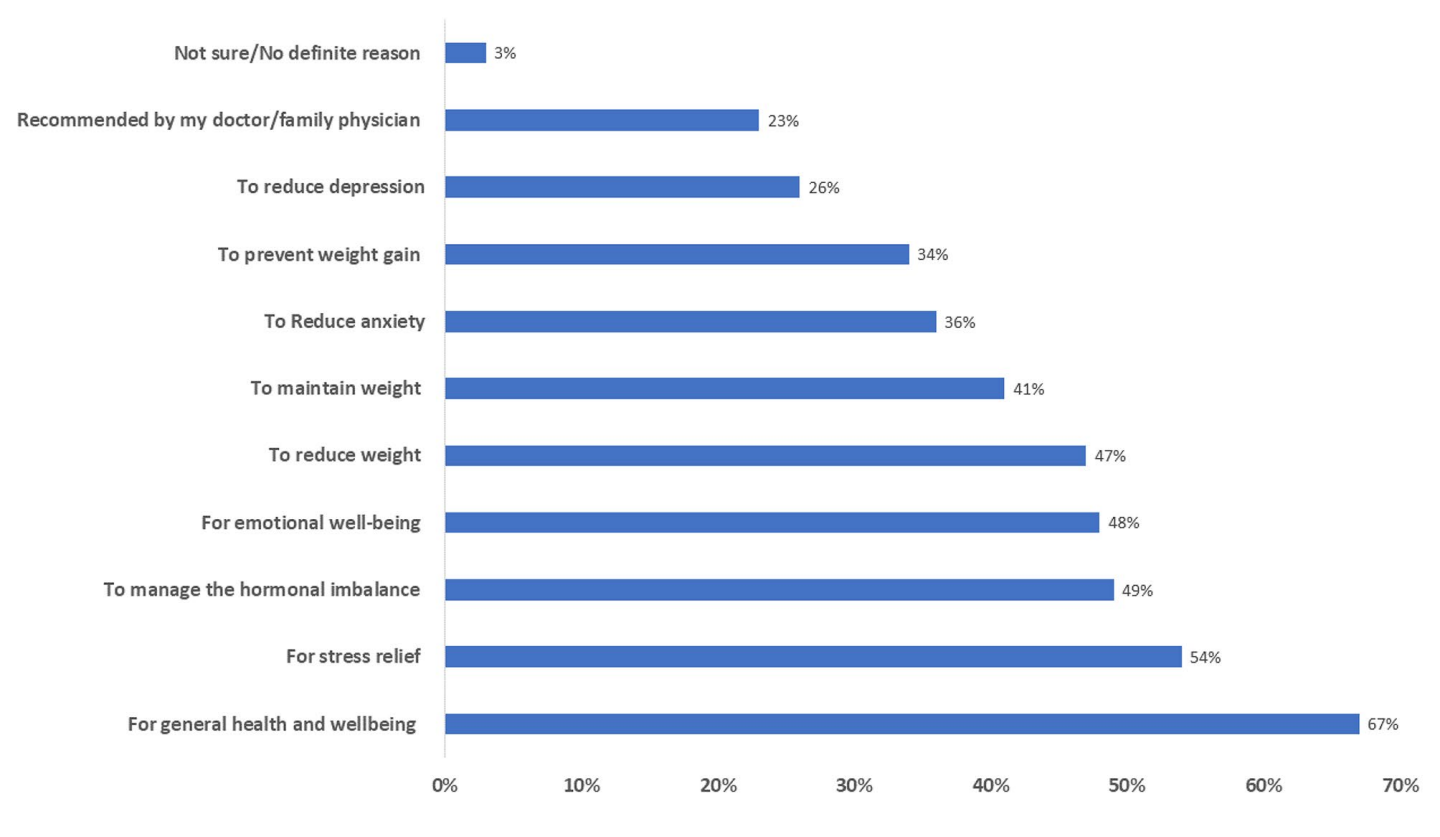
Reasons for practising yoga reported by the participants (n = 1679)

As shown in Fig. 3, reasons for practising yoga included; helping to promote general health and well-being (1197/1679,67%), overall weight management (64%), stress management (54%), balancing hormones (49%), emotional well-being, (48%), to reduce anxiety (36%), to reduce depression (26%) and due to recommendation by family physician/doctor (23%). On a scale of 0–10, we asked women about their recommendation and helpfulness of yoga to manage symptoms of PCOS. Almost two-thirds (n = 1224/1696,72%) scored six and above for recommendation to their family/friends with a median of 8.0 (IQR = 4) (Supplemental File 2, Table 4).

### Reasons for not using yoga and future interest

Among women who never used yoga, when asked about their reasons for not practising yoga, lack of motivation (650/1107, 59%), lack of time (39%) and inflexibility (31%) were commonly reported (Table 5). Other reasons were: ‘unable to find the right yoga teacher’ (23%), and ‘do not feel fit enough’ (23%). However, 81% (998/1238) expressed future interest in using it to manage symptoms of PCOS, whereas 12% were neutral and only 7% were disinterested.

**Table 5:** Reasons for non-utilization of yoga and future interest, (N = 1355)

Reasons for not practising yoga	(n = 1107)
Lack of motivation (to practice at home or attend classes)	650 (59)
Lack of time (to practice yoga at home or attend classes)	433 (39)
Do not feel flexible enough	353 (31)
Can not find the right yoga teacher	256 (23)
Do not feel fit enough	253 (23)
Feel embarrassed about my body	202 (18)
Do not feel strong enough	199 (18)
Financial (not enough money to pay for yoga classes)	175 (16)
Distance (yoga place is far from me)	114 (10)
Physical barriers such as injury	49 (4)
Other than the above (please specify)	93 (8)
Missing	248 (18)
**Interested in trying yoga in future**	(n = 1238)
Interested	998 (81)
Neither interested nor disinterested	158 (12)
Not at all interested	82 (7)
Missing	14 (1)

## Discussion

This novel and the large international study provide evidence on the extent of use of TCIM, types, reasons for use and non-use, perceived effectiveness, and adverse events in women of Indian ethnicity with PCOS. We demonstrated that most ethnic Indian women with PCOS use TCIM (80%) regardless of their age, occupation, education, relationship status, BMI, and family history of PCOS and T2D. However, those living in India use TCIM more frequently compared to those living outside India. Most women reported using two to three types of TCIM modalities (52%). They frequently use their indigenous health practices such as yoga (57%) and Ayurveda (37%), which is not uncommon given the ethnic, cultural, and traditional reasons associated with the high use of TCIM. Women use these modalities not only for their overall health and well-being but also to help manage specific symptoms of PCOS (such as menstrual irregularities). Despite reporting several barriers to the use of Ayurveda and yoga, there was a high interest in using these therapies in the future among non-users which needs to be further investigated.

To date, there have been limited studies on the use of TCIM in women with PCOS. Only one study conducted by Arentz et al. in 2012 (n = 493) reported on the prevalence of the use of complementary medicine in women with PCOS living in Australia (70%) [7]. However, in our study, the prevalence of TCIM is 80%, a little higher compared to Arentz et al’s study. This difference could be because of several reasons: Arentz et al. focused on the use of naturopathy, western herbal medicines, vitamins, and supplements rather than on the traditional medicines approaches that are indigenous to different cultures. Moreover, our study is of a large sample size, focused on women of Indian ethnicity and used strict eligibility criteria and diverse recruitment strategies compared to that of Arentz et al. Therefore, we provide a unique insight into the use of TCIM modalities in ethnic Indian women where the use of traditional medicines is culturally rooted, institutionally recognized, and practised.

Previous studies conducted among the non-PCOS population have confirmed associations between various demographic factors such as age and education and the use of TCIM [35–37]. However, except for the place of residency no other demographic factor was found to be a significant predictor of TCIM usage in our study. Notably, variations in participant characteristics between our study and previous studies may account for this difference. We found that women living in India had increased odds of using TCIM (AOR 1.81 (1.43 to 2.28)) compared to those women living outside of India. This is not surprising because, TCIM modalities are also well-recognised, regulated and commonly used for primary health needs in India [38]. For example, an Ayurveda doctor gets trained in a University-based institution for 5.5 years and then might choose to do a postgraduate or a PhD of (3–6 years) in a particular Ayurvedic department [18]. However, it is important to notice that a considerable number of Indian women living outside India reported using TCIM in our study 73% (n = 536/734) which is almost two times compared to a previous study conducted in South Africa in Indian communities (38.5%) [25]. This large difference could be attributed to the fact that the previous study was conducted general population and our study focuses on women with PCOS. Furthermore, migrants tend to use their native TCIM practices in their migrant country before or at least alongside their use of conventional medicines [39]. While doing so, they might face several barriers in a migrant country where it may not be well-recognised and regulated [40] which might impact greatly on their help-seeking behaviour. Therefore, our findings have implications for health professionals providing care to ethnic Indian women with PCOS irrespective of their geographical location because there is still a reasonably high proportion and future interest in using TCIM in our sample. Further in-depth qualitative studies exploring use of TCIM in ethnic Indian women with PCOS living outside India are warranted.

The drivers of TCIM use are similar across the globe and within cultures, especially among Asian groups where deeply rooted cultural values, a recommendation from family/friends, dissatisfaction with the current medical approach, perception of TCIM being natural and safe, and the quest for holistic health encourage people to use various TCIM modalities [35, 41, 42]. We found similar trends in our study. Additionally, we found that women are using/preferring various TCIM modalities not just for general health and well-being but also to manage symptoms of PCOS. For example, in our study, there were higher odds of using TCIM by women with menstrual irregularity concerns and married women living in India with a history of pregnancy and taking treatment to conceive. Additionally, most women used yoga for weight management and psychological and emotional health related to PCOS. Almost half of them (58%) preferred a PCOS-specific yoga class. We also found that women in our study were recommended to practice yoga by their family doctors/physicians (23%), which adds to the growing acceptance of yoga therapy among health professionals [43]. This highlights the importance of creating awareness and equipping them with the right information about the role of yoga in ethnic Indian women with PCOS. Thus, there is a possibility that women might shift between different health approaches not just between conventional medicines and TCIM but also among the TCIM modalities as per their health needs, convenience and beliefs as demonstrated in our study. It is, therefore, important that these women are treated considering their social and cultural choices about healthcare.

Concurrent use of TCIM with other modalities/conventional medicine has been documented in previous studies of drug interactions [26, 44]. Addressing this issue was out of the scope of our study, however, we found that the use of Ayurveda as ‘an additional therapy alongside other treatments’ was one of the reasons for using Ayurveda (12%). Although we are unsure if women consumed Ayurvedic medicines or followed diet/lifestyle aspects of Ayurveda, it still imposes a risk since TCIM are perceived as “natural” and “safe to use” and patients may not be aware of these potential implications. Thus, we support the need for informed TCIM treatment decisions and demonstrate the need for in-depth understanding to examine the concurrent use of TCIM and other forms of TCIM/drugs by women with PCOS. Additionally, a patient-centred collaboration between conventional and TCIM practitioners might help to understand and explore the role of culturally competent integrative care for women with PCOS to meet their specific health needs and to provide safe treatments [45].

Although perceived to be safe by most women, 9% of women reported experiencing some adverse events (minor) such as gastrointestinal issues such as acidity, heavy periods and increased body heat while using Ayurveda medicines. These reported events were mostly related to the perception of increased body heat in the body. Body heat is a general phenomenon Indian women describe in the context of their illness and medicine [46]. Sensations of heat and cold in the body are perceived to be caused by illness or because of ingesting medicines. There are several myths attached to the use of traditional medicines. Previous studies have examined these cultural norms and tried to understand the concepts behind these expressions [46, 47]. Therefore, we suggest that Ayurveda clinicians should carefully ask the patients about their perceptions of Ayurvedic medicines, interpret these expressions properly and take this opportunity to explain, collect, report, and monitor any adverse event.

### Implication for policy, practice, and research

PCOS is a complex disorder, requiring a multidisciplinary approach, and women are likely to consult TCIM practitioners alongside conventional healthcare providers. Based on the findings of our paper, we recommend that researchers, policymakers, and health professionals should carefully evaluate the interest and pattern of use of TCIM in ethnic Indian women with PCOS, help drive more research into these practices, translate the research into clinical settings and support these women to make informed decisions. This is particularly important because of the increased prevalence of PCOS in this population, and the evidence of high use of TCIM which we have demonstrated. A step towards multidisciplinary care (practitioners of conventional and TCIM-yoga) for women with PCOS has been initiated in India which is reported to be feasible and culturally sensitive [48]. However, our study highlights that there is yet a lot more to be done to provide safe and evidence-based TCIM practices in terms of the exchange and transformation of knowledge between the health practitioners of different modalities, and the need for the right infrastructure and skills to deliver the best care to these women.

Another point we would like to highlight from our findings is the necessity to understand and explore the regulation of TCIM outside India as we found a considerable number of women with PCOS living outside India are using these modalities. Considering current trends in globalization and migrations, there is a need to consider the role of global statutory regulation for different TCIM modalities including Ayurveda and yoga. For example, in Australia, Ayurveda practice is not yet well-regulated which can potentially risk the patient-safety and un-informed TCIM use [40]. While the integration of TCIM practices into healthcare globally is an identified need [49], our study findings suggest that more efforts are needed in education, research and policy to implement these strategies into practice. Therefore, interprofessional collaboration in different country settings among key stakeholders is crucial in establishing the long-term clinical and research infrastructure to guide TCIM use in women of a certain ethnicity with PCOS.

### Strengths and limitations

This study has several strengths. By using several recruitment channels including Facebook, Instagram, and Twitter, along with posting to specific groups relevant to the study and using paid advertisements on social media, we were able to reach a large and diverse random sample of ethnic Indians living both within and outside of India. We demonstrated that a large online international survey is achievable. To the best of our knowledge, this is one of the largest datasets of ethnic Indian women with PCOS. As the survey was voluntary and anonymous and no monetary benefits were given to the participants for participating in the study, the results are likely to reflect the authentic views of women with PCOS in the community. We also followed strict inclusion criteria where we excluded women who reported that they were not diagnosed with PCOS by a conventional medical doctor. However, we are not able to confirm which diagnostic criteria of PCOS were used to diagnose them as this was out of the scope of our study. Nonetheless, the results may be generalisable to women with PCOS, as our data are likely to reflect the authentic views of ethnic Indian women with PCOS living in the community.

Our findings do have some limitations. Firstly, we did not design the study as a case-control study which limits the comparisons with non-Indian women. Secondly, our findings are limited by the sampling framework, since women who have not sought support through electronic social media, eliminate the potential to reach women with PCOS who are not active online. Limitations also include the exclusion of women who were younger than 18 years of age and older than 55 years of age and women who were not able to read and write English well. PCOS continues to affect women in their adolescence and post-menopause, however, their experiences were not captured in this survey. This study also might have a risk of response bias although we tried to minimize this risk while designing the study and by piloting the questionnaire among the consumers. It is important to note that there could be cultural and religious reasons behind the high use of yoga and Ayurveda in our study sample, however, we cannot confirm this because we did not obtain such information. Future qualitative enquiry into the high use of TCIM in PCOS would help to strengthen these findings. And, finally, we could not determine non-response and selection bias due to the online nature of our survey.

## Conclusion

Ethnic Indian women with PCOS use various TCIM modalities including their indigenous health systems such as yoga and Ayurveda to manage their symptoms of PCOS. Given the complexities involved in PCOS, TCIM approaches may play an important role in the ongoing treatment regimen to help provide culturally sensitive patient-centred care. These women have unique health needs and preferences for treatment methods, moreover, the complementary nature of the TCIM attracts them to use it for symptoms that are not managed by current medical approaches. Therefore, this study is a crucial step in understanding views and patterns of the use of TCIM in ethnic Indian women to help support them in making informed decisions.

### Electronic supplementary material

Below is the link to the electronic supplementary material.

Supplementary Material 1

Supplementary Material 2

## Data Availability

The data and materials used to support the findings of this paper are included in this article, however, the complete datasets used and/or analysed during this study are available from the corresponding author on reasonable request.

## Abbreviations

TCIM: Traditional, complementary, and integrative medicine
PCOS: Polycystic Ovary Syndrome
IQR: Interquartile ranges
I-CAM-Q: International Complementary and Alternative Medicine Questionnaire
SD: Standard deviations
T2D: Type 2 diabetes
AOR: Adjusted odds ratio
CI: 95% confidence interval
