# Supplementary material for: Use of traditional and complementary medicine by ethnic Indian women living with polycystic ovary syndrome: a global survey
Source: BMC Complement Med Ther. 2023 Nov 3;23:392. doi: 10.1186/s12906-023-04229-9 (PMC10623873; doi:10.1186/s12906-023-04229-9)
Supplement: Supplementary file 1 — Supplementary Material 1 [file 12906_2023_4229_MOESM1_ESM.docx]

Polycystic Ovary Syndrome (PCOS) in ethnic Indian women: a global survey

**Before we begin, let's make sure that you are eligible for this survey.**
 
Do you identify yourself as a woman of Indian ethnic ancestry? (Have at least one parent or grandparent who was born in India)

- Yes (1)
- No (2)

Have you been diagnosed with PCOS by a medical doctor?

- Yes (1)
- No (2)

What is your age in years?

▼ 18 (1) ... 55 (38)

What is your current body weight in kilograms? [(u](https://www.calculator.net/weight-calculator.html?fromnumber=80&amp;unitFrom=pound&amp;unitTo=kilogram&amp;x=57&amp;y=16)[se this convertor if needed to convert pounds to kilograms)](http://www.calculator.net/weight-calculator.html?fromnumber=80&amp;unitFrom=pound&amp;unitTo=kilogram&amp;x=57&amp;y=16)

________________________________________________________________

What is your height in centimetres? [(use th](https://www.calculatorsoup.com/calculators/conversions/heightftcm.php)[is convertor if needed to convert feet and inches to centimetres)](http://www.calculatorsoup.com/calculators/conversions/heightftcm.php)

________________________________________________________________

|  |
| --- |

Which of the following health conditions do you have other than PCOS? (multiple answers)

- Anxiety (1)
- Chronic fatigue (2)
- Coronary artery disease (myocardial infarction/heart attacks and/or angina) (3)
- Depression (4)
- Eating disorder (5)
- Endometrial cancer (6)
- High blood pressure (7)
- High cholesterol levels or triglyceride levels (8)
- Sleep disorders (eg. sleep apnea) (9)
- Thyroid related problems (eg. hypothyroid, hyperthyroid) (10)
- Type 2 diabetes (11)
- ⊗I don’t have any other health condition other than PCOS (12)
- Other than above (please specify) (13) ________________________________________________

Which of the following immediate family members have been diagnosed with PCOS? (multiple answers)

- Mother (1)
- Sister (2)
- Daughter (3)
- ⊗None of the above (4)

Which of the following immediate family members have been diagnosed with type 2 diabetes? (multiple answers)

- Brother (1)
- Daughter (2)
- Father (3)
- Mother (4)
- Sister (5)
- Son (6)
- ⊗None of the above (7)

**Now, please share your experience of diagnosis and signs and symptoms of PCOS.**   How old were you when you first experienced the signs and symptoms of PCOS?

▼ Less than 10 (1) ... 55 (47)

How old were you when you first visited a doctor for any of the above signs and symptoms?

▼ Less than 10 (1) ... 55 (47)

At what age were you diagnosed with PCOS?

▼ Less than 10 (1) ... 55 (47)

 Which country you were living in when you were diagnosed with PCOS?

▼ Afghanistan (1) ... Zimbabwe (195)

Who performed the diagnosis?

- Cardiologist (1)
- Dermatologist (2)
- Endocrinologist (3)
- Gynecologist/obstetrician (4)
- General practitioner/family physician/family doctor (5)
- Infertility specialist (6)
- Psychiatrist (7)

Which of the following medical treatment methods have you used to manage PCOS? (multiple answers)

- Anti-androgen drugs (to correct male-hormone levels) (1)
- Anti-obesity drugs (2)
- Bariatric surgery (3)
- Combined oral contraceptive pills (estrogen + progestin) (4)
- Intrauterine device (IUD) (eg. Mirena or Depo Provera) (5)
- Intrauterine insemination (IUI) (6)
- In-vitro fertilization (IVF) or intracytoplasmic sperm injection (ICSI) (7)
- Laparoscopic surgery (ovarian drilling) (8)
- Metformin (insulin-sensitizing medicines) (9)
- Ovulation induction to fall pregnant (eg. Letrozole, Clomid, gonadotropins) (10)
- Never used any modern medications for PCOS (11)
- Other than above (please specify) (12) ________________________________________________

Which of the following signs and symptoms of PCOS concern you the most? **(Please select any three)**

- Acne/pimples (1)
- Anxiety (2)
- Cysts on the ovaries (in an ultrasound) (3)
- Depression (4)
- Difficulty losing weight (5)
- Excess unwanted hair growth over face (6)
- Excess hair loss (7)
- High blood levels of androgens/male hormones (eg. testosterone) (8)
- Increased metabolic risk (eg. fear of developing type 2 diabetes) (9)
- Increased tendency for weight gain (10)
- Irregular menstrual cycles/periods (11)
- Problems with ovulation (12)
- Not able to fall pregnant (Infertility) (13)
- Other than above (please specify) (14) ________________________________________________

**Now we would like to ask you about any traditional and complementary medicine (T&CM) that you may have used to manage PCOS.** Please tell us which of the following traditional health systems you have tried to manage PCOS?

- Acupuncture (1)
- Ayurveda (2)
- Chiropractic (3)
- Homeopathy (4)
- Naturopathy (5)
- Osteopathy (6)
- Siddha Medicines (7)
- Unani Medicines (8)
- Western herbal medicines (9)
- Traditional Chinese medicines (10)
- ⊗Never used any of the T&CM (11)
- Other than above (please specify) (12) ________________________________________________

Skip To: Q27 If Now we would like to ask you about any traditional and complementary medicine (T&CM) that you may... != Ayurveda (Traditional Indian Medicine)

Skip To: End of Block If Now we would like to ask you about any traditional and complementary medicine (T&CM) that you may... = Ayurveda (Traditional Indian Medicine)

What are your reasons for not choosing Ayurvedic medicines? (multiple answers)

- Lack of scientific evidence (1)
- Poor quality of herbal medicines (2)
- Not prescribed or recommended by my doctor (3)
- Not recommended by family/friends (4)
- Not enough information (5)
- Not covered by my insurance (6)
- Not easily available (7)
- Not confident to use in conjunction with medical drugs (8)
- Takes too long to work (9)
- Smell or taste of the preparations (10)
- Not sure if it will work (11)
- ⊗Not sure/no definite reason (12)
- Other than above (please specify) (13) ________________________________________________

In future, how interested you would be in trying Ayurvedic diet or medicines to manage PCOS?

- Not interested at all (1)
- Slightly interested (2)
- Neither interested nor disinterested (3)
- Very interested (4)
- Extremely interested (5)

**You indicated that you have used Ayurvedic medicine (Traditional Indian Medicine) to manage PCOS, please tell us more about it.**

 When did you first visit an Ayurvedic practitioner for PCOS related management?

- In the last 6 months (1)
- In the last 1 year (2)
- In the last 1-5 years (3)
- More than 5 years (4)

How long have you been using Ayurveda medicine to manage PCOS?

- From past 6 months (1)
- Past 1 year (2)
- Past 1-5 years (3)
- More than 5 years (4)

What or who most influenced your decision to use Ayurvedic medicine? (multiple answers)

- Allied health professional (eg. dietician, exercise physiologist) (1)
- Family/friends/neighbors/colleagues (2)
- Internet (3)
- Magazine or newspaper (4)
- Medical doctor/fertility specialist (5)
- Self (6)
- Television (7)
- Another complementary medicine practitioner (eg. naturopath, homeopath) (8)
- Other than above (please specify) (9) ________________________________________________

What are the reasons for choosing Ayurvedic medicine? (multiple answers)

- Agree with its principles (1)
- Easily available (2)
- Cheaper/affordable (3)
- Has multiple benefits (4)
- Has natural ingredients (5)
- Helps in overall health and well-being (6)
- Lack of success with trying other treatment (7)
- Recommended by friends/family (8)
- Safe and no side effect (9)
- Traditionally accepted (10)
- Using as additional therapy alongside other treatment (11)
- Other than above (please specify) (12) ________________________________________________

What forms of Ayurvedic treatment you have tried to manage the symptom of PCOS? (multiple answers)

- Ayurvedic lifestyle (eg. Ayurvedic diet/regimen) (1)
- Ayurvedic medicines for internal consumption (2)
- Ayurvedic medicines for external applications (3)
- Panchakarma (eg. Vamana, Virechana, Basti, Nasya) (4)
- Other than above (please specify) (5) ________________________________________________

Have you ever had a negative reaction or unwanted side effects to Ayurvedic medicines?

- Yes (1)
- No (2)
- Unsure (3)

Skip To: Q35 If Have you ever had a negative reaction or unwanted side effects to Ayurvedic medicines?  = Yes

Skip To: Q36 If Have you ever had a negative reaction or unwanted side effects to Ayurvedic medicines?  = No

Skip To: Q36 If Have you ever had a negative reaction or unwanted side effects to Ayurvedic medicines?  = Unsure

Please write what were those negative reaction or unwanted effects.

________________________________________________________________

________________________________________________________________

________________________________________________________________

________________________________________________________________

________________________________________________________________

On a scale of 0-10, how likely are you to recommend Ayurveda to your family members or friends to manage PCOS?

- 0 (0)
- 1 (1)
- 2 (2)
- 3 (3)
- 4 (4)
- 5 (5)
- 6 (6)
- 7 (7)
- 8 (8)
- 9 (9)
- 10 (10)

Have you ever practised yoga since your diagnosis of PCOS?

- Yes (1)
- No (2)

Skip To: End of Block If Have you ever practised yoga since your diagnosis of PCOS? = Yes

Skip To: Q38 If Have you ever practised yoga since your diagnosis of PCOS? = No

What are your reasons for not choosing yoga? (multiple answers)

- Cannot find the right yoga teacher (1)
- Distance (yoga place is far from me) (2)
- Do not feel flexible enough (3)
- Do not feel fit enough (4)
- Do not feel strong enough (5)
- Financial (not enough money to pay for yoga classes) (6)
- Feel embarrassed about my body (7)
- Lack of time (to practice yoga at home or attend classes) (8)
- Lack of motivation (to do practice at home or attend classes) (9)
- Physical barriers such as injury (10)
- Other than above (please specify) (11) ________________________________________________

In future, how interested you would be in practicing yoga to manage PCOS?

- Not at all interested (1)
- Slightly interested (2)
- Neither interested nor disinterested (3)
- Very interested (4)
- Extremely interested (5)

**You indicated that you have used yoga to manage PCOS, please tell us more about it.**
  
 How long have you been practising yoga?

- Last 6 months (1)
- Last 1 year (2)
- Last 1-5 year (3)
- More than 5 years (4)

What were your specific reasons for practising yoga? (multiple answers)

- For general health and wellbeing (1)
- For emotional well-being (2)
- For stress relief (3)
- Recommended by my doctor/family physician (4)
- To manage hormonal imbalance (5)
- To maintain weight (6)
- To prevent weight gain (7)
- To reduce weight (8)
- To reduce anxiety (9)
- To reduce depression (10)
- ⊗Not sure/No definite reason (11)
- Other than above (please specify) (12) ________________________________________________

Which type/style of yoga have you been practising since your diagnosis of PCOS? (multiple answers)

- Ashtanga (1)
- Bikram /Hot yoga (2)
- Iyengar (3)
- Krishnamacharya tradition or (Viniyoga) (4)
- Kundalini (5)
- Mixed style (6)
- Power Yoga (7)
- Shivananda Yoga/Yoga Vidya (8)
- Traditional Hatha Yoga (9)
- Unsure about the style (10)
- Vinyasa (11)
- ⊗Whatever is available (12)
- Other than above (please specify) (13) ________________________________________________

On average, how often you currently practice yoga?

- Daily (1)
- 5-6 times per week (2)
- 3-4 times per week (3)
- 1-2 times per week (4)
- Twice a month (5)
- Once in a month (6)
- Less than once a month (7)
- I don’t practice yoga anymore (8)

Skip To: Q46 If On average, how often you currently practice yoga? = I don’t practice yoga anymore

Skip To: Q46 If On average, how often you currently practice yoga? = I don’t practice yoga anymore

Where do you practise yoga? (multiple answers)

- Home (1)
- Workplace (2)
- Yoga studio/school/institute (3)
- Fitness center/gym (4)
- Park or other public outdoor location (5)
- Other than above (please specify) (6) ________________________________________________

What elements generally involves in your yoga practise?  (multiple answers)

- Asana (physical postures) (1)
- Dhyana (meditation) (2)
- Mantra chanting (3)
- Pranayama (breathing practices) (4)
- Yama/Niyama (5)
- Yogic diet (6)
- Yogic kriya (eg. jala neti, vamana dhouti) (7)
- Other than above (please specify) (8) ________________________________________________

What type of yoga practise would you prefer?  (multiple answers)

- Individual one-on-one (1)
- Group practice (2)
- General yoga class (3)
- Yoga class designed to manage symptoms of PCOS (4)
- Other than above (please specify) (5) ________________________________________________

Which of the following mode of instruction would you prefer to practise yoga? (multiple answers)

- Face to face sessions (1)
- Live online sessions (2)
- Pre-recorded online sessions (eg. internet, social media, DVD, TV) (3)
- Anything which is available/mixed (4)
- Other than above (please specify) (5) ________________________________________________

Are there any challenges or barriers that prevent you from participating in yoga?

- Yes (1)
- No (2)
- Not sure (3)

Skip To: Q49 If Are there any challenges or barriers that prevent you from participating in yoga? = Yes

Skip To: Q50 If Are there any challenges or barriers that prevent you from participating in yoga? = No

What are those challenges? (multiple answers)

- Cannot find the right yoga teacher (1)
- Distance (yoga place is far from me) (2)
- Do not feel flexible enough (3)
- Do not feel fit enough (4)
- Do not feel strong enough (5)
- Financial (not enough money to pay for yoga classes) (6)
- Feel embarrassed about my body (7)
- Lack of time (to practice yoga at home or attend classes) (8)
- Lack of motivation (to do practice at home or attend classes) (9)
- Physical barriers such as injury (10)
- Other than above (please specify) (11) ________________________________________________

On a scale of 0-10, how likely are you to recommend yoga to your family members or friends to manage PCOS?

- 0 (0)
- 1 (1)
- 2 (2)
- 3 (3)
- 4 (4)
- 5 (5)
- 6 (6)
- 7 (7)
- 8 (8)
- 9 (9)
- 10 (10)

**Finally, please tell us a few general things about yourself. Please note that this is an anonymous survey and your answers will be kept confidential.**

 In which country were you born?

▼ Afghanistan (1) ... Zimbabwe (195)

Q64 In which country do you currently reside?

▼ Afghanistan (1) ... Zimbabwe (195)

What is the highest level of school you have completed or the highest degree you have received?

- No formal education (1)
- Lower secondary (2)
- Upper secondary (3)
- Post-secondary non tertiary education (eg. vocational/ apprenticeship/ certificate) (4)
- First stage of tertiary education (not leading directly to an advanced research qualification) (5)
- Second stage of tertiary education (leading to an advanced research qualification) (6)
- Undergraduate degree (bachelor’s degree) (7)
- Postgraduate (master’s degree/PhD/post-doctoral degree) (8)

What is your current relationship status?

- Divorced (1)
- In a relationship/ de facto (2)
- Married- living with a partner (3)
- Single (4)
- Widowed (5)
- Separated (6)

Which statement best describes your current occupation? (multiple answer)

- Employed full time (1)
- Employed part-time (2)
- Home duties/ caring for family or children (3)
- Retired (4)
- Self-employed/Freelance (5)
- Studying (eg. going to school/college/university) (6)
- Unemployed- Looking for work (7)
- Unemployed- not looking for work (8)
- Unable to work because of symptoms/treatment of PCOS (9)
- Volunteer work (10)
- Other (please specify) (11) ________________________________________________

Have you ever been pregnant?

- Yes (1)
- No (2)

Skip To: End of Block If Have you ever been pregnant? = No

Have you ever needed any treatments to help you become pregnant?

- Yes (1)
- No (2)

How many biological children do you have?

- None (1)
- Currently Pregnant (2)
- 1 (3)
- 2 (4)
- More than 2 (5)

Finally, is there anything else you would like to share about your experience of PCOS?

________________________________________________________________
