# Supplementary material for: Use of traditional and complementary medicine by ethnic Indian women living with polycystic ovary syndrome: a global survey
Source: BMC Complement Med Ther. 2023 Nov 3;23:392. doi: 10.1186/s12906-023-04229-9 (PMC10623873; doi:10.1186/s12906-023-04229-9)
Supplement: Supplementary file 2 — Supplementary Material 2 [file 12906_2023_4229_MOESM2_ESM.docx]

**Supplemntal File 2**

**Supplemental Table 1: Association between history of pregnancy and taking treatment and the use of TCIM**

| **Predictors (Independent variables)** | **TCIM use (yes/no^a^)** | | | |
| --- | --- | --- | --- | --- |
|  | **Odds ratio^b^** | **95% Wald Confidence Interval** | | **P-value^c^** |
|  |  | **Lower** | **Upper** |  |
| Country of current residence-= India | 2.698 | 1.747 | 4.166 | **<0.001** |
| Country of current residence-= Outside India | Ref |  |  |  |
| Highest Education category =Undergraduate | 1.093 | .446 | 2.680 | .845 |
| Highest Education Category = Postgraduate | 1.038 | .674 | 1.600 | .865 |
| Highest Education Category below = Undergraduate | Ref |  |  |  |
| Current relationship =Divorced/widowed/separated | 1.333 | .290 | 6.133 | .712 |
| Current relationship =Married/in a relationship | 3.062 | 1.210 | 7.749 | **0.018** |
| Current relationship =Single | Ref |  |  |  |
| Women with a history of pregnancy and taking treatment to conceive =Yes | 1.597 | 1.036 | 2.461 | **0.034** |
| Women with a history of pregnancy and taking treatment to conceive =No | Ref |  |  |  |

^a^Reference value

^b^Multivariable binary logistic regression analysis adjusted for the factors presented in the table

^c^Significant P value is marked in bold

**Supplemental Table 2: Association between the presence of the top three key concerns and the use of TCIM**

| **Top 3 key concerns of PCOS** | **Total participants (n=3130)^a^** | **TCIM**  **yes** | **TCIM**  **no** | **P value^b^** | **OR (95% CI)^c^** |
| --- | --- | --- | --- | --- | --- |
| **Irregular menstrual cycle/periods** |  |  |  |  |  |
| Yes | 1971 (63) | 1630 (83) | 341 (17) | **<0.001** | 1.41 (1.16 to 1.71), **<0.001** |
| No | 1153 (37) | 879 (76) | 274 (24) |  | Ref |
| **Difficulty losing weight** |  |  |  |  |  |
| Yes | 1808 (58) | 1439 (80) | 369 (20) | 0.237 | - |
| No | 1316 (42) | 1070 (81) | 246 (19) |  |  |
| **Excess facial hair growth** |  |  |  |  |  |
| Yes | 1451 (46) | 1162 (80) | 289 (20) | 0.787 | - |
| No | 1673 (54) | 1347 (81) | 326 (19) |  |  |

^a^Missing values= 6

^b^Chi-square test

^c^Binary logistic multivariable model, adjusted for age, BMI, birth country, residence country, education, relationship, and occupation

Significant values are marked in bold

**Supplemental Table 3: Adverse events while using Ayurveda as reported by the participants**

| **Category** | **List of negative reactions or unwanted effects commonly reported by the participants (n=97)** |
| --- | --- |
| Gastrointestinal Tract (GIT) | Acidity= 8  Bloating/gaseous feeling=7  Diarrhoea=6  Nausea/vomiting= 6  Mouth soreness= 2  Stomach ache= 2  Irritable bowel=1  Miscellaneous= 3 |
| Skin and Hair | Acne= 9  Hair fall= 3  Skin irritation= 2 |
| Weight | Weight gain=5  Weight loss= 2 |
| Menstruation | Heavy periods= 12  Period pain=2  Skipped periods= 1 |
| Other | Heat in the body =15  Mood swing=2  Breast size increased=1  Dizziness=1  Reduced immunity=1 |

**Supplemental Table 4: Recommendation of Ayurveda and yoga to family/friends to manage symptoms of PCOS**

| **Participant’s score^s^** | **Recommendation for Ayurveda, (n=1090)** | **Recommendation for yoga, (n= 1696)** |
| --- | --- | --- |
| 0 | 50 | 36 |
| 1 | 23 | 20 |
| 2 | 35 | 19 |
| 3 | 47 | 50 |
| 4 | 45 | 50 |
| 5 | 226 | 297 |
| 6 | 85 | 110 |
| 7 | 112 | 207 |
| 8 | 145 | 295 |
| 9 | 109 | 222 |
| 10 | 213 | 390 |

^a^Participant’s scores on a scale of 0-10, where 0 indicates extremely negative opinion and 10 indicates extremely positive opinion (n)
